# Convalescing Mandibular Anterior Crowding through Piezocision and the Micro-Osteoperforation Surgical Procedure—A Clinical Comparative Study

**DOI:** 10.3390/jpm14020173

**Published:** 2024-01-31

**Authors:** Raghunath Nagasundara Rao, Karuna Elza Oommen, Raghavendra Reddy Nagate, Mohammed A. Al-Qarni, Abdul Razzaq Ahmed, Shreyas Tikare, Shankar T. Gokhale, Ahmed A. AlBariqi, Mohamed Fadul A. Elagib, Saurabh Chaturvedi

**Affiliations:** 1 Department of Orthodontics & Dentofacial Orthopedics, JSS Dental College & Hospital, JSS Academy of Higher Education & Research, (Deemed to Be University), Mysuru 570015, India; dr.nraghunath@jssuni.edu.in (R.N.R.); karunaoommen92@gmail.com (K.E.O.); 2 Department of Periodontics and Community Dentistry, College of Dentistry, King Khalid University, Abha 62529, Saudi Arabia; tikane@kku.edu.sa (S.T.); sgokhale@kku.edu.sa (S.T.G.); aaalbarqi@kku.edu.sa (A.A.A.); mfdel@kku.edu.sa (M.F.A.E.); 3 Department of Restorative Dentistry, College of Dentistry, King Khalid University, Abha 61471, Saudi Arabia; maalqarny@kku.edu.sa; 4 Department of Prosthetic Dentistry, College of Dentistry, King Khalid University, Abha 62529, Saudi Arabia; aamoshtag@kku.edu.sa; 5 Department of Dental Research Cell, Dr. D. Y. Patil Dental College and Hospital, Dr. D. Y. Patil Vidyapeeth, Sant-Tukaram Nagar, Pimpri, Pune 411018, India

**Keywords:** piezocision, micro-osteoperforation, accelerated orthodontics, lower anterior decrowding, orthodontic, periodontology, oral surgery

## Abstract

Background: Minimally invasive periodontic (perio) surgical procedures, piezocision, and micro-osteoperforation are useful techniques for accelerating tooth movement. These techniques also offer advantages in the orthodontic (ortho) and aesthetic domains. This study aimed to evaluate and compare the rates of lower anterior decrowding with piezocision and micro-osteoperforation. Methods: This clinical study included 24 patients requiring fixed orthodontic treatments. Two periodontic techniques (piezocision (PZ) and micro-osteoperforation (MOP)) were considered for the orthodontic treatments. Each patient was randomly allocated to either the piezocision (PZ) group or the micro-osteoperforation (MOP) group. The piezocision group received five radiographically guided incisions on the labial surface of the alveolar bone, whereas the micro-osteoperforation group received one to three MOPs each using a mini-implant drill between the six lower anterior teeth, and later, an initial arch wire was ligated to each bracket. Little’s irregularity index (LII) was calculated using a digital vernier caliper on study models every four weeks until decrowding was achieved. The difference in the rates of lower anterior crowding between the piezocision and micro-osteoperforation groups was analyzed to determine the statistical significance. Results: The rates of irregularity index change during decrowding were 4.38 ± 0.61 in the piezocision group and 3.82 ± 0.47 in the micro-osteoperforation group. Piezocision was found to be 1.2 times faster than micro-osteoperforation in terms of the rate of decrowding. Conclusion: The advanced perio–ortho combination technique was advantageous in accelerated decrowding. In comparison to MOP, there was an increase in the rate of decrowding with PZ. Decrowding can be completed quickly with PZ, and it can thus be used to treat crowding effectively in a limited time frame.

## 1. Introduction

In the last few decades, the need for adult orthodontic treatment has increased. However, orthodontic treatments in adults, children, and adolescents are not usually the same. The treatment in adult patients is mainly focused on dentoalveolar compensation [1,2,3,4]. Patients, particularly adults seeking orthodontic treatment, are continually requesting shorter treatment durations without any additional discomfort and with outstanding results. In orthodontic therapy, diminishing treatment durations and reducing side effects without losing treatment efficacy have become difficult tasks. According to Tayer’s research [5], 33% of adult patients declined to receive orthodontic treatment when considering the length of the procedure as well as the discomfort and difficulty of wearing orthodontic appliances. The lengthy duration of treatment is a significant concern for adult orthodontic patients. Adults typically demand more aesthetic appliances and shorter treatment times [6,7].

Treatment goals are typically achieved within 18–24 months, according to common wisdom. Long-term orthodontic treatments increase the risks of dental caries, white spot lesions, periodontal problems, external apical root resorption, gingivitis, periodontitis, and TMJ difficulties and causes reductions in patient compliance [4,8,9,10]. Researchers are considering new techniques to minimize treatment times without sacrificing treatment efficacy because of the desire for shorter treatment durations.

Orthodontic force’s mechanical impact on periodontal ligament cells triggers the production of osteoclasts through the RANK-RANKL pathway (receptor activator of NF-kB (RANK) and receptor activator of NF-kB ligand (RANKL)) and the release of cytokines, prostaglandins, and other chemical messengers [11,12]. Accelerated orthodontic tooth movement (AOTM) using various techniques has led to a reduction in the duration of treatment. It has been found to be associated with regional acceleratory phenomena (RAP), through which tissue forms at a faster rate than the normal local regeneration process. Frost [13] described the regional acceleratory phenomenon (RAP) as a “complex reaction of mammalian tissues to various noxious stimuli”.

A different approach for the acceleration of tooth movement was used by Wilcko and Wilcko [14] in 2001. The acceleration of tooth movement that they focused on was due to the RAP. The RAP (decalcification/recalcification of the alveolus), which was a physiological idea, replaced the mechanical concept of a “bony block movement” relating to the corticotomy effect. The periodontally accelerated osteogenic orthodontics (PAOO) approach was developed by the Wilcko brothers, who additionally added bone grafts after decortication and applied orthodontic forces one week before the procedure [6].

A reduction in decrowding time can be achieved using various acceleration procedures like pharmacological methods; physical methods like lasers and magnets; and periodontic surgical techniques like corticotomy (COT), piezocision, micro-osteoperforations, etc.

Two periodontic techniques—piezocision and micro-osteoperforation—are less invasive when compared to other surgical techniques like Wilkodontics and corticotomy. The acceptance of corticotomies is still low because both clinicians and patients consider them to be too invasive [15,16,17]. To solve this issue, a new minimally invasive surgical technique known as piezocision was created. It was first introduced in 2009 by Dibart [18], who fused the flapless corticision method with the benefit of grafting provided by PAOO. A piezoelectric knife is used to decorticate the alveolar bone and start the regional accelerator phenomena; this procedure incorporates buccal gingival microincisions. This minimally invasive procedure permits bone deficiency or gingival recession correction through the selective tunneling of hard or soft tissue grafts.

The piezoelectric knife has been used to create microincisions in the buccal cortex, and it has a very precise and selective cutting action; hence, it preserves the integrity of roots. Micro-osteoperforation is one of the safest and least invasive surgical techniques by which transmucosal perforations are made within the alveolar cortex near the region of the desired tooth movement. Micro-osteoperforation, sometimes referred to as alveocentesis, is a cutting-edge method for accelerating tooth movement with little to no surgical intervention. As there are no flap elevations or incisions made before the osteoperforation, it is a less invasive technique. A tool that can be used to make MOPs is called Propel (Propel Orthodontics, USA). Theoretically, this approach works by amplifying inflammatory markers that are typically released during orthodontic tooth movement [19,20]. This, in turn, speeds up tooth movement.

Various studies have been conducted individually assessing piezocision and micro-osteoperforation, but to the best of our knowledge, not enough consideration was given to comparing these two AOTM techniques. We conducted a literature search for the past 10 years (2010–2023) and found that no study has compared the PZ and MOP techniques (Table 1).

Thus, the present clinical study was conducted to evaluate and compare the efficacy of piezocision and micro-osteoperforation in lower anterior decrowding using a conventional Mclaughlin, Bennet, and Trevisi (MBT) bracket prescription. The null hypothesis was that there would be no difference between these two techniques in their efficacy in alleviating lower anterior crowding.

## 2. Materials and Methods

### 2.1. Study Design and Study Participants

The study protocol was developed, and ethical clearance was obtained from the institutional ethics committee of JSS Dental College and Hospital (date—25 September 2019; JSSDCH IEC 54/2019). The chief researcher recruited patients needing fixed orthodontic treatments from the outpatient department of the Department of Orthodontics and Dentofacial Orthopedics of JSS Dental College and Hospital, Mysore.

The patients for this study were selected based on strict inclusion and exclusion criteria. The criteria for inclusion were as follows: (i) patients requiring fixed orthodontic treatments within the age group of 14 to 25 years, (ii) patients exhibiting moderate-to-severe crowding with Little’s irregularity index values ˃5 mm, (iii) healthy periodontal tissues, and (iv) the absence of systemic diseases. The exclusion criteria were as follows: (i) patients with systemic diseases; (ii) patients who had taken antibiotics, corticosteroids, or calcium channel blockers for a long time; (iii) gingivitis; (iv) periodontally compromised patients; and (v) radiographic or clinical evidence of bone loss. Each patient included in this study was asked to sign an informed consent form, and the study was explained in detail.

### 2.2. Group Division

A total of 50 patients agreed to be part of this study. Based on the inclusion criteria, 26 patients were excluded, and the remaining 24 patients were randomly divided into two groups: group I—PZ and group II—MOP.

**Figure 1:**
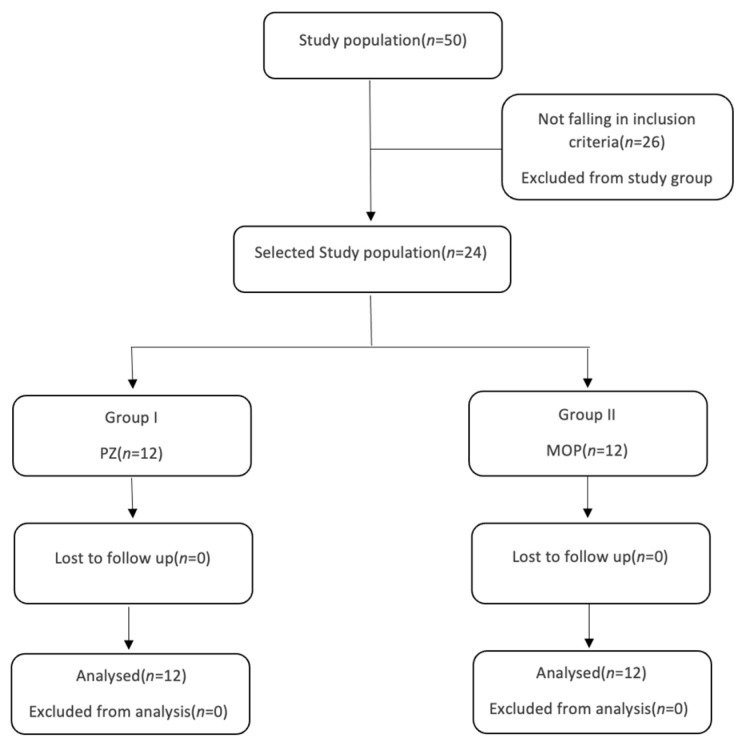
Flow diagram of patient recruitment and follow-up [PZ—Piezocision procedure; MOP—Micro-osteoperforation procedure].

The piezocision group and the micro-osteoperforation group consisted of 12 subjects each with conventional MBT bracket prescriptions. A single operator performed the periodontal surgery procedure. Extraction of the first premolar tooth under local anesthesia was advised before starting the fixed orthodontic treatment. Both groups were bonded with MBT prescription appliances with 0.022-inch slots after extraction (Figure 1).

### 2.3. Periodontic Procedures

#### 2.3.1. Piezocision Procedure (PZ)

**Figure 2:**
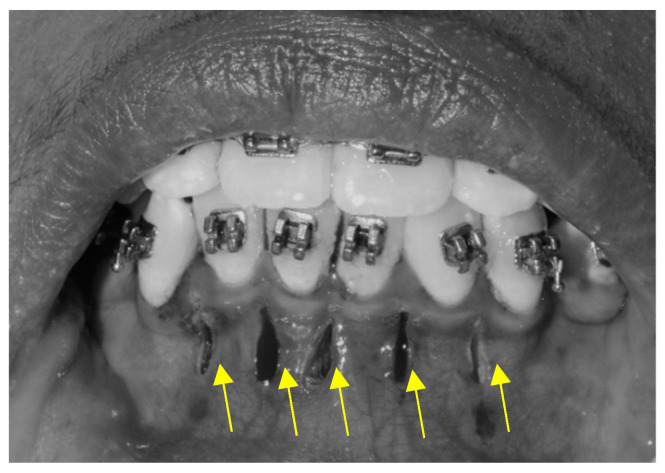
Piezo incisions were made in the labial cortex of lower anterior teeth, as shown by yellow arrows.

**Figure 3:**
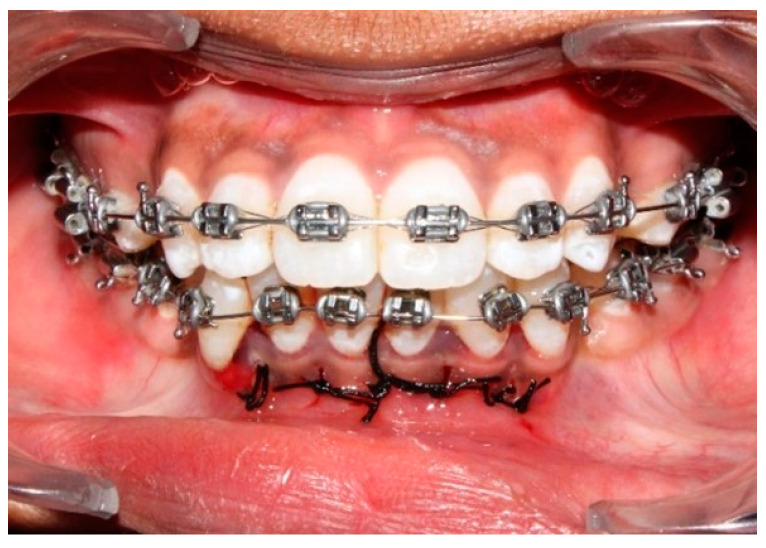
Interrupted sutures given after piezocision procedure.

In the piezocision procedure, five interproximal vertical microincisions through the periosteum and below the interdental papilla were made between the six anterior teeth using a No. 15 blade. The incisions were made approximately 5 mm apical to the mesial and distal interdental papilla, while adjacent tooth roots were used as a reference (Figure 2). Cortical alveolar incisions of approximately 2 mm were made using a BS-1 piezo surgery knife. The incisions were sutured with non-resorbable 4–0 silk in an interrupted pattern (Figure 3).

#### 2.3.2. Micro-Osteoperforation Procedure (MOP)

**Figure 4:**
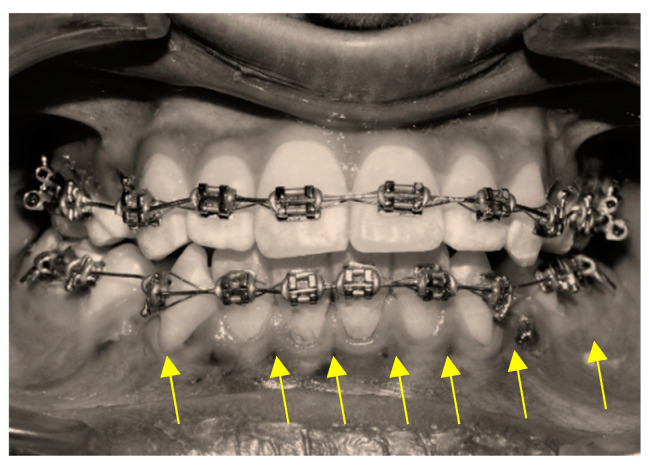
MOPs were made in the labial cortex of lower anterior teeth, as shown by yellow arrows.

The MOP procedure was performed without raising the flap, and the alveolar bone between the six lower anterior teeth was cut along the labial surface using 2–3 micro-osteoperforations (MOPs) (Figure 4). An orthodontic mini-implant drill bit (1.2 mm) with a contra-angle hand driver was used to make the perforations. To keep the depth of the MOPs consistent, the mini-implant drill bits were guarded with a stop.

### 2.4. Orthodontic Procedure

**Figure 5:**
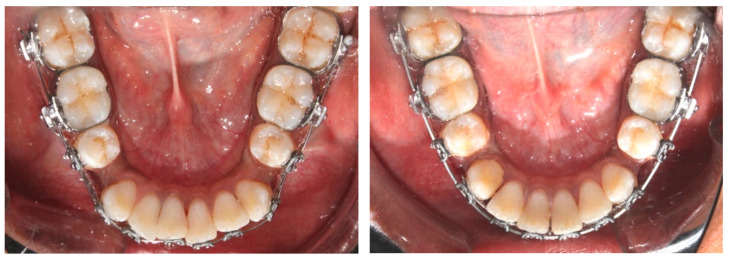
Intraoral photographs at T0 and T2.

The initial arch wire (0.014” NiTi) was ligated to each bracket during the same appointment at which piezocision or MOP was performed. Arch wires were replaced when the next wire could be inserted into the bracket slots with a minimal amount of deflection. The sequence of arch wires was 0.014 NiTi, 0.016 NiTi, 0.016 × 0.022 NiTi, 0.017 × 0.025 NiTi, 0.019 × 0.025 NiTi, and 0.019 × 0.025 SS. The patients were recalled the next day to check for any procedure-related complications. The chief researcher measured Little’s irregularity index (LII) every 4 weeks, and this study was considered finished when LII was less than 1 mm (Figure 5). Also, the total alignment time required for lower anterior decrowding was evaluated.

We also measured LII on study models taken at monthly intervals:

- T0—at the initiation of orthodontic treatment.
- Tx—at the termination of the decrowding stage when LII was less than 1 mm.
- x denotes the month in which final decrowding was achieved.

**Figure 6:**
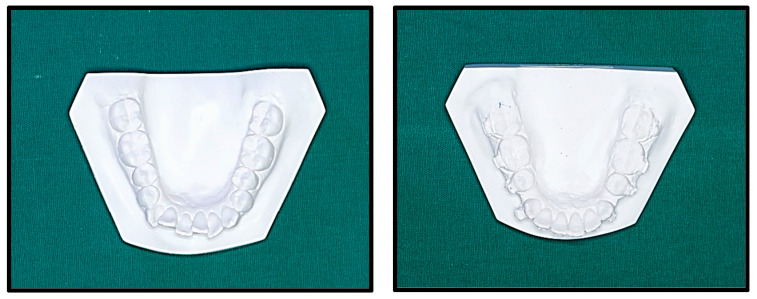
Study models at T0 and T2.

An alginate impression was taken at each stage, and the impression was poured with dental plaster (calcium sulphate). The cast was labeled with the patient’s outpatient number and date (Figure 6).

### 2.5. Statistical Methods

A prior power calculation was performed for this study based on pilot study results. The sample size was estimated to be 10 subjects per group using the formula $n=\frac{2(S^{2})}{d^{2}}{\left[z_{1-\alpha /2}+z_{1-\beta }\right]}^{2}$ to obtain 80% power in the trial. Statistical analysis was performed by SPSS version 22.0 for windows, and descriptive statistics were used. For the descriptive statistics, the mean and standard deviation were used. An independent *t*-test was used for inferential statistics to compare the results of the assessment stages between the 2 groups.

## 3. Results

The total number of samples assessed in this study was 24 (*n* = 12 each for the PZ and MOP groups) (Table 2). The patients consisted of 8 males and 16 females with a mean age of 19.6 ± 2.6 years.

**Figure 7:**
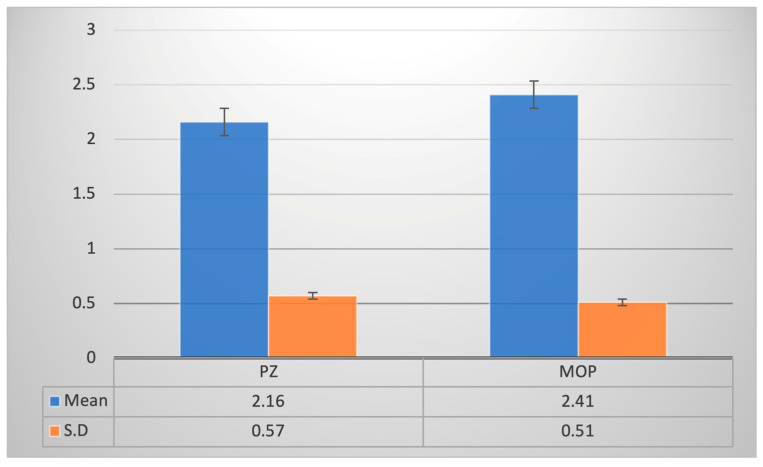
Time taken for decrowding, piezocision (PZ), micro-osteoperforation (MOP).

The maximum time taken for decrowding was 3 months in both the piezocision group and the micro-osteoperforation group. The minimum time taken for decrowding was 1 month in the piezocision group and 2 months in the micro-osteoperforation group (Table 2). The mean time taken for decrowding was 2.16 ± 0.57 months in the piezocision group and 2.41 ± 0.51 months in the micro-osteoperforation group (Figure 7). The time taken in the piezocision group was 0.25 months/7.5 days less than the time taken in the micro-osteoperforation group, and it was statistically insignificant (*p* ˃ 0.05) (Table 3).

The maximum rates of irregularity index change during decrowding were 5.2 and 4.8 in the piezocision group and micro-osteoperforation group, respectively (Table 2). The minimum rates of irregularity index change during decrowding were 3.4 and 3.2 in the piezocision group and micro-osteoperforation group, respectively (Table 2).

**Figure 8:**
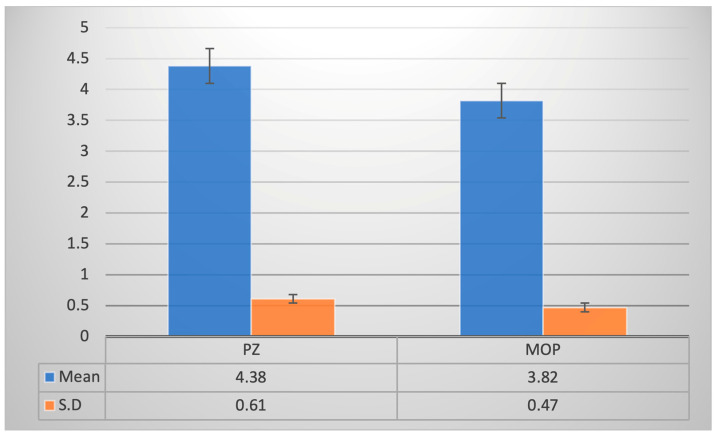
Rate of decrowding.

The mean rates of irregularity index change during decrowding were 4.38 ± 0.61 in the PZ group and 3.82 ± 0.47 in the MOP group, respectively (Figure 8), which were observed to be statistically significant (*p* ˂ 0.05) (Table 4). The rate of decrowding in the PZ group was 0.6 mm/month greater than that in the MOP group. PZ was 1.2 times faster than MOP in terms of the rate of decrowding.

## 4. Discussion

One of the most common types of malocclusions is dental crowding. Conventional treatment methods differ between extraction and non-extraction procedures. AOTM has been desired for its various advantages like reduced treatment duration, fewer complications, and increased patient satisfaction. The additional benefit of this procedure is that it causes reduced root resorption because of decreased cortical bone resistance together with slow relapse.

The gradual remodeling of alveolar bone causes orthodontic tooth movement (OTM). A potential mechanism to accelerate the biologic response is to injure bone, thereby accelerating the normal physiologic processes involved in wound healing. They start with initial osteoclastic activity followed by osteoblastic activity, which increase bone density. Once the repair process begins, the cytokine activity around the tooth increases, thus enhancing the rate of tooth movement during orthodontic therapy [13].

Fixed orthodontic treatment comprises a variety of tooth movements that work together to achieve the best possible occlusion. Tissue reaction occurs around the affected tissues during orthodontic therapy on a chemical, biological, and mechanical basis. Orthodontic tooth movement is characterized as a balanced process due to bone resorption on the compression side and bone deposition on the tension side. Many methods have been devised to shorten the length of orthodontic treatment by accelerating tooth movement. Whether the relationship between increased RANKL and decreased OPG concentrations with higher tooth movement in rats can be replicated in humans is one of the main research questions.

In piezoelectric surgery, ultrasonic micro-vibrations only cut brittle mineralized tissues, and soft tissues are preserved. A piezoelectric device enables safe and accurate osteotomies without any osteonecrotic injury due to its micrometrical and selective cut [50,51]. Additionally, excessive force is not necessary in piezoelectric surgery, suturing is advised everywhere to reduce scarring, and patient pain is relatively low. The grafting option and the short operation time are additional benefits of this technique. Flap elevation is not performed, which further reduces the length of the procedure and the amount of postoperative discomfort. The disadvantage of this method is that the cuts must be made blindly because there is no flap reflection. To prevent injury to the roots, navigation or surgical stents are thought to be helpful. Other disadvantages include the minimal cutting of the bone; therefore, the expected duration of the RAP effect might not be achieved. Because it is a blind procedure, it is essential to plan the positions of the MOPs to protect the roots. It is not possible to graft either hard or soft tissues to strengthen and repair the periodontium during the surgery. The thick cortex in the mandible makes this time-consuming and requires repeated procedures, which increases treatment costs and chair time [6,18,19].

A study by Omar Gibrael examined the effectiveness of piezocision in expediting the decrowding of lower anterior teeth. The study group noted a reduction of 59% for the overall treatment time in the PZ group compared to the conventional control group. It was found that piezocision caused decrowding acceleration and reduced the overall time by about 59%, and the overall average alignment time was 53.5 ± 12.5 days. The result of the present study shows that the average time taken for decrowding acceleration was 2.16 ± 0.57 months, equivalent to 60 ± 3 days [52]. Charavet et al., in their study, demonstrated a significant reduction of 43% in the overall treatment time in the PZ group when compared to the conventional group [53]. In the present study, the overall time taken for decrowding was 10.3% less in the PZ group when compared to the MOP group. The difference in the rate may be attributed to the fact that the present study included cases with moderate-to-severe crowding and the use of the MBT system, compared to the earlier study where they included cases with mild-to-moderate crowding and the use of the Damon self-ligating system. Mustafa et al. compared the efficiency of PZ and decisions in patients with moderate crowding using accelerated orthodontic treatment strategies. The time required for orthodontic treatment in a piezocision group with a self-ligating system was decreased by 27% compared to a conventional treatment with self-ligating brackets [54]. This was reasonably similar to the present study, where the duration was 10.3% shorter in the PZ group. Flavio Uribe et al. compared the duration of mandibular decrowding using PZ and conventional orthodontics and concluded that there was no significant difference in the rate of alignment or the duration of treatment for mandibular crowding when PZ-assisted orthodontics were used compared to conventional orthodontics. The time required to correct decrowding was around 10 days less in the piezocision group, but this was statistically insignificant [55]. In the present study, the time taken for decrowding was 7.5 days less in the PZ group compared to the MOP group. The cases in that study group mostly had mild anterior crowding, and non-extraction treatments were performed. This offers a plausible explanation for the decreased rate of decrowding noted in that study. In our study, patients had moderate-to-severe crowding; hence, they required extraction, probably increasing the RAP effect.

Azaitun Akma Shahrin et al. examined the efficiency of micro-osteoperforation in the decrowding of anterior teeth. The rate of alignment in the MOP group was 2.12 mm higher in the first month, peaked at 3.01 mm in the second month, and decreased thereafter [56]. This agreed with our study, where the rate of irregularity index change was 3.82 ± 0.47. Mehak Bansal et al. investigated mini-implant-facilitated MOP’s effectiveness in speeding up mandibular anterior teeth decrowding. The rate of alignment in the MOP group was around 2.13 times faster at the first recall visit, but the rate decreased at the further appointments compared to the conventional group [57]. In a previous study, authors compared piezocision and conventional orthodontic treatment in terms of mandibular alignment. It was found that the piezocision group showed 1.6 times faster decrowding in the first 4–5 weeks compared to the conventional group. Also, the total time required was 20 days less in the piezocision group compared to the control group [58]. This was consistent with the current investigation’s findings, where piezocision was 1.2 times faster.

Basema et al. compared the efficiency of piezocision and MOP in accelerating orthodontic tooth movement. The rate of canine retraction was assessed for three months in both groups. Piezocision accelerated the pace of tooth movement compared to micro-osteoperforation, but the overall net movement was not statistically significant [58].

PZ and MOP techniques of accelerated orthodontic treatment can be used with aligners, as presented in a few cases [44,59]. In orthodontics, aligner therapy has become an established treatment option. At an orthodontic clinic, many patients specifically want treatment with aligners. The amount of time required for each aligner sequence to be worn and its cleaning and maintenance with disinfectant are disadvantages of this treatment [60]. The use of aligners with AOTM is not very common. Hannequin et al. presented a case report of a Class III malocclusion that was treated ortho-surgically with corticotomies using a piezoelectric knife for rapid presurgical decompensation and clear aligners, followed by a mandibular sagittal split osteotomy. A patient-managed smartphone application called “dental monitoring” was used to manage the clinical follow-up of aligner-mediated tooth movement, in which aligners were changed every four days. This allowed for the early detection and correction of even the smallest orthodontic movement faults. The authors provided an excellent example of how to employ dental monitoring equipment when there is a need for extremely close observation due to a rapid rate of tooth movement [44]. In 2023, Pérez et al. conducted a study relating orthodontic aligner treatment and RANKL and osteoprotegerin (OPG) concentrations in crevicular fluid as markers of bone remodeling and found no statistically significant differences in crevicular fluid RANKL or OPG concentrations between treated and control teeth or between adjustments every 7 or 14 days [61].

While providing accelerated orthodontic treatment, precautions should be taken in terms of the systemic health of patients, especially for patients receiving bisphosphonates. Bisphosphonates are thought to have an impact on orthodontic therapy and tooth movement because they alter bone metabolism. As a result of their inhibition of osteoclastic activity, they lessen bone resorption. Orthodontic tooth movement promotes alveolar bone turnover and may improve the local absorption of bisphosphonates. On the other hand, frequent bisphosphonate administration combined with orthodontic movement may produce an even more enhanced cycle of increased local uptake and release of the active medication. Therefore, more prospective randomized clinical trials must be carried out to obtain more reliable scientific data regarding the effect of bisphosphonates on both traditional and rapid orthodontic tooth movement [62].

The limitations of the present study include its small sample size. It is recommended to include patients with mild, moderate, and severe crowding and to analyze the effects of PZ and MOP in each of the subsegments separately. An assessment of periodontal parameters was not taken into consideration. An assessment of periodontal changes would provide insight into the effect of the treatment on the soft tissues surrounding teeth. The movement of teeth due to orthodontic forces not only affects the hard bone tissue but also affects the soft tissues. In the present study, while we educated the patients about related soft tissue changes, we did not assess them, which is an important limitation of this study. Also, the present study did not evaluate the effects of herbal or synthetic mouthwashes or any associated drugs [63].

## 5. Conclusions

Within the limitations of this study, it can be concluded that lower anterior teeth decrowding can be performed at a faster rate with the piezocision technique of accelerated orthodontics. The results of this study indicated that the rate of alignment with micro-osteoperforation was significantly lower than with PZ. Additionally, it was noticed that in terms of the pace of decrowding, piezocision was 1.2 times faster than micro-osteoperforation. With PZ, decrowding can be finished quickly, and it can thus be used to treat crowding effectively in a limited time frame. Further studies are recommended to take periodontal parameters into consideration and analyze larger samples.

## Figures and Tables

**Table 1:** Related studies conducted in previous years (2010–2021).

Year of Study	Author(s) of Publication	Test Subject	Sample Size	Presence of CG	Study Type	Procedure Being Assessed
2010	Sanjideh et al. [21]	A	Five	×	SM	COT
2010	Teixeira et al. [22]	A	Forty-Eight	√	-	Soft tissue flap and osteoperforations
2011	Keser and Dibart [23]	H	One	×	-	PZ
2013	Keser and Dibart [24]	H	One	×	-	PZ
2013	Alikhani et al. [25]	H	Twenty	√	CO	MOP
2014	Murphy et al. [26]	A	Forty-Four	√	-	Corticision
2014	Milano et al. [27]	H	One	×	-	PZ
2014	Dibart et al. [28]	A	Ninety-Four	√	-	PZ
2016	Murphy et al. [29]	A	Forty-Four	√	-	Corticision
2016	Aksakalli et al. [30]	H	Ten	×	SM	PZ
2016	Charavet et al. [31]	H	Twenty-Four	√	CO	PZ
2016	Abbas et al. [32]	H	Twenty	×	SM	COT, PZ
2016	Dibart et al. [33]	A	Two Hundred Seventy-Six	√	-	piezoelectric knife, bur, handheld screw device
2017	Uribe et al. [34]	H	Twenty-Nine	√	CO	Piezotome corticisions
2018	Sugimori et al. [35]	A	Fifty	√	O	MOP
2018	Alkebsi et al. [36]	H	Thirty-Two	√	CO	MOP
2018	Chan et al. [37]	H	Twenty	√	CO	MOP
2018	Attri et al. [38]	H	Sixty	√	CO	MOP
2019	Hou et al. [39]	H	One	×	-	PZ
2019	Strippoli et al. [25]	H	Twenty-Four	√	CO	PZ
2019	Van Gemert [40]	A	Thirteen	×	SM	MOP
2019	Sivarajan et al. [41]	H	Thirty	×	SM	MOP
2019	Charavet et al. [42]	A	Sixty	√	O	PZ
2020	Hatrom et al. [39]	H	Twenty-Six	√	CO	PZ
2020	Khlef et al. [43]	H	Forty	×	-	COT, PZ
2020	Hannequin et al. [44]	H	One	×	-	COT, PZ
2021	Hatrom et al. [45]	H	Twenty-Three	√	CO	PZ
2021	Alvarez et al. [46]	H	Thirty-Six	√	CO	PZ
2021	Kernitsky et al. [47]	A	Eighteen	√	-	piezoelectric decortications
2021	Sharon et al. [25]	H	Thirty	√	CO	MOP
2022	Charavet C et al. [48]	A	Sixty	×	-	PZ
2023	Battista et al. [49]	A	Twenty-Two	√	-	piezoelectric knife/rotary bur

A—animal study; H—human study; CG—control group; SM—split-mouth design; O—orthodontic force; CO—conventional orthodontics; COT—corticotomy; PZ—piezocision; MOP—micro-osteoperforations. √ = Presence of CG, × = Absence of CG, - = Study type no mentioned.

**Table 2:** Data for PZ and MOP groups.

Sample No.	T0	Tx	T0 − Tx	Time (Interval) Taken for Decrowding (T) in Months	Rate of Decrowding (T0 − Tx/T)
PZ1	10.7	0.5	10.2	3	3.4
PZ2	11.1	0.8	10.3	3	3.43
PZ3	9.7	0.5	9.2	2	4.6
PZ4	8.6	0.4	8.2	2	4.1
PZ5	10.2	0.3	9.9	2	4.95
PZ6	9	0.6	8.4	2	4.2
PZ7	10.7	0.3	10.4	2	5.2
PZ8	11.6	0.2	11.4	3	3.8
PZ9	5.6	0.4	5.2	1	5.2
PZ10	9.2	0	9.2	2	4.6
PZ11	9.8	0.2	9.6	2	4.8
PZ12	8.8	0	8.8	2	4.4
MOP1	9.2	0.9	8.3	2	4.15
MOP2	7.2	0.4	6.8	2	3.4
MOP3	7.6	0.4	7.2	2	3.6
MOP4	10.5	0.3	10.2	3	3.4
MOP5	8.8	0.4	8.4	2	4.2
MOP6	9.8	0.2	9.6	2	4.8
MOP7	10.4	0.2	10.2	3	3.4
MOP8	11.4	0	11.4	3	3.8
MOP9	8.5	0.3	8.2	2	4.1
MOP10	9.6	0	9.6	3	3.2
MOP11	11.1	0.3	10.8	3	3.6
MOP12	8.6	0.2	8.4	2	4.2

**Table 3:** Independent-sample *t*-test between groups for the time taken.

		*t*-Test for Equality of Means		
		*t*	df	Sig. (Two-Tailed)
Time Taken	Equal variances assumed	−1.119	22	0.275

**Table 4:** Independent-sample *t*-test between groups for the rate of decrowding.

		*t*-Test for Equality of Means		
		*t*	df	Sig. (Two-Tailed)
Rate	Equal variances assumed	2.518	22	0.020

## Data Availability

The data can be made available on demand by the chief researcher for academic purposes by email.
